# Medicated Gutta-Percha Canal Points

**Published:** 1903-04-15

**Authors:** L. T. Canfield


### MEDICATED GUTTA-PERCHA CANAL POINTS.

---

BY L. T. CANFIELD.

---

An ideal root-filling must contain properties which shall eliminate the predisposition to septic changes, after devitalization, and present unsuitable soil for bacterial development. It should be germicidal, penetrating, non-irritating, not contracting, and impermeable to everything that may be found free in the oral cavity. With some the only reason for putting anything into a pulpless tooth is to keep the food out. This may be all right, but why fill a root at all if it be not to exclude bacteria which makes filling a necessity. The aim of the surgeon to-day is asepsis rather than antisepsis —to avoid infection rather than disinfection.

After two years of experimenting I am convinced that gutta-percha, combined with wood creosote, formaldehyde, iodoform and oil of cassia, makes an antiseptic and ideal root-canal filling.

Every dentist knows the value of wood creosote for infected tissue. It is antiseptic, slightly anesthetic, penetrating and healing. Iodoform is anesthetic, penetrating, persistent, and antiseptic. It crystallizes on the walls of the canal chambers and remains continuously active. Formaldehyde is used for its remarkable drying and hardening properties together with its great antiseptic value. To so combine these germicides and antiseptics that they might not lose their medicinal properties and forever remain active, was by no means an easy task. There are so many complicating conditions in the mouth that the relative value of stoppings to resist the passage of bacteria must be determined in the laboratory. To do this the conditions must be as near as possible the same as exist in the mouth. A long list of tabulated experiments show bacterial growths in different root-canal stoppings from nearly every known method of root-canal filling. Tubes filled with the medicated gutta-percha are the only ones which show no infection or bacterial development. I have used these medicated points in pulpless teeth under all conditions and have never had acute inflammation.

After deciding on the germicides for medication, the next step was the thorough incorporation of the medicines with the gutta-percha, so that the medicinal properties would not be lost but forever remain active. This was accomplished after nearly a year of experimentation and the gutta-percha formed into cone-shaped points of assorted lengths and sizes. The advantages of these medicated antiseptic points are that the tubules as well as the foramen are sealed thus preventing infiltration of infective secretions into the dentine, making putrescence utterly impossible and leaving no fertile soil for bacterial development. The tooth can not, therefore, absorb and retain septic matter, thus preserving the color, and every molecule of the filling is impregnated with these lasting germicides which do not lose their medicinal qualities as do all root-canal dressings by being absorbed.

I have filled with the medicated gutta-percha badly decayed and infected roots, in the mouth, for the express purpose of extracting for experiment, after six months of perfect immunity from trouble to the patient. I removed and placed in the incubator found no trace of bacterial development, then removed the filling from root, and made a chemical analysis, finding all the germicidal and medicinal qualities intact. These experiments have been carried on in different forms by me for the past three years, and I have the tables and data which I invite any and all to see at any time.

